# Examining resting state functional connectivity and frequency power analysis in adults who stutter compared to adults who do not stutter

**DOI:** 10.3389/fnhum.2024.1338966

**Published:** 2024-02-05

**Authors:** Atefeh Valaei, Sobhan Bamdad, Arsalan Golfam, Golnoosh Golmohammadi, Hayat Ameri, Mohammad Reza Raoufy

**Affiliations:** ^1^Department of Linguistics, Tarbiat Modares University, Tehran, Iran; ^2^Department of Biomedical Engineering, Faculty of Engineering, Shahed University, Tehran, Iran; ^3^Department of Speech Therapy, School of Rehabilitation Sciences, Semnan University of Medical Sciences, Semnan, Iran; ^4^Department of Physiology, Faculty of Medical Sciences, Tarbiat Modares University, Tehran, Iran; ^5^Institute for Brain Science and Cognition, Faculty of Medical Science, Tarbiat Modares University, Tehran, Iran

**Keywords:** functional connectivity, frequency power, stuttering, resting state, adult

## Abstract

**Introduction:**

Stuttering is a speech disorder characterized by impaired connections between brain regions involved in speech production. This study aimed to investigate functional connectivity and frequency power during rest in adults who stutter (AWS) compared to fluent adults (AWNS) in the dorsolateral prefrontal cortex (DLPFC), dorsolateral frontal cortex (DLFC), supplementary motor area (SMA), motor speech, angular gyrus (AG), and inferior temporal gyrus (ITG).

**Materials and methods:**

Fifteen AWS (3 females, 12 males) and fifteen age- and sex-matched AWNS (3 females, 12 males) participated in this study. All participants were native Persian speakers. Stuttering severity in the AWS group was assessed using the Persian version of the Stuttering Severity Instrument Fourth Edition (SSI-4). Resting-state electroencephalography (EEG) was recorded for 5 min while participants sat comfortably with their eyes open. We analyzed frequency band power across various frequency bands and investigated functional connectivity within the specified speech region.

**Results:**

Significant between-group differences were found in band powers including alpha, beta, delta, theta, and gamma, specifically in the premotor, SMA, motor speech, and frontal regions. AWS also showed increased coherence between the right motor speech region compared to controls. We demonstrate that the proposed hierarchical false discovery rate (FDR) method is the most effective for both simulations and experimental data. In the expected regions, this method revealed significant synchrony effects at an acceptable error rate of 5%.

**Conclusion:**

The results highlight disrupted functional connectivity in AWS at resting state, particularly in speech-related and associated areas. Given the complex neurological basis of developmental stuttering, robust neural markers are closely linked to this phenomenon. These markers include imbalanced activity within brain regions associated with speech and motor functions, coupled with impaired functional connectivity between these regions. The cortico-basal ganglia-thalamo-cortical system governs the dynamic interplay between cortical regions, with SMA as a key cortical site. It is hypothesized that the aberrant resting state functional connectivity will impact the language planning and motor execution necessary for fluent speech. Examining resting-state metrics as biomarkers could further elucidate the neural underpinnings of stuttering and guide intervention.

## Introduction

Stuttering is a speech fluency disorder that affects more than 80 million people worldwide (Yairi and Ambrose, 2004). It is characterized by disruptions in the normal flow of speech, often resulting in repetition, prolongation, or complete blocking of sounds or words. Approximately 5–8% of preschool children exhibit stuttering behaviors, and about 1% of the population continues to stutter into adulthood (Bloodstein and Bernstein-Ratner, 2008). Despite extensive research, the precise underlying neural underpinnings of stuttering remain unclear. Various techniques have been used to study the neural correlates of stuttering. Functional magnetic resonance imaging (fMRI) studies have examined functional connectivity patterns in adults who stutter (AWS), revealing widespread connectivity differences compared to fluent speakers, though findings are inconsistent. For instance, reduced functional connectivity in specific brain regions and increased functional connectivity within speech-associated regions have been reported (Xuan et al., 2012; Shojaeilangari et al., 2021). Some studies find certain connectivity patterns correlate with stuttering severity (Lu et al., 2012) or improve after interventions (Yang et al., 2016). Still, the heterogeneous results across fMRI investigations underscore the need for further research into brain functional dynamics in AWS.

Critically, fMRI measures slow blood-oxygen-level dependent signals, lacking the temporal precision to characterize moment-to-moment neural activity relevant for resting state and speech production (Glover, 2011). Therefore, we aimed to use electroencephalography (EEG) to capture rapid neural dynamics in AWS. EEG provides millisecond temporal resolution to measure transient state-dependent functional connectivity abnormalities hypothesized in stuttering. Despite lower spatial resolution, EEG’s accessibility and affordability enabled efficient examination of temporal brain dynamics linked to stuttering pathophysiology. EEG precisely measures electrical activity from neuronal populations to study dynamics during speech and rest. This electrophysiological approach is key for investigating the neurophysiological processes underlying stuttering. Specifically, EEG studies have revealed abnormal power in certain frequency bands during speech production and non-speech oral motor tasks in AWS compared to fluent controls. While previous research has mainly focused on speech-motor areas, stuttering is increasingly seen as a neurological timing deficit that affects broader sensorimotor networks (Yang et al., 2016; Etchell et al., 2018). This is consistent with converging evidence suggesting that stuttering involves widespread abnormalities in brain function and connectivity, extending beyond just speech-motor regions (Chang et al., 2018).

However, the extent of resting state functional connectivity anomalies in AWS across the entire brain remains unclear. Additionally, while spectral power, which reflects synchronized neural oscillations, provides complementary insights into intrinsic network dysfunction (Doesburg et al., 2016), it has been less investigated in AWS compared to children who stutter. For instance, EEG records have revealed atypical timing of auditory-motor integration during speech planning and execution in people who stutter (Beal et al., 2011). Studies have shown reduced EEG coherence within key networks such as the default mode, salience, and frontal-parietal networks in adults who stutter compared to fluent controls (Chang and Zhu, 2013). Furthermore, spectral power analyses of resting state EEG have elucidated elevated beta and gamma power in frontal and motor regions (Joos et al., 2014).

Within the central nervous system realm, AWS often exhibits atypical regulation of the articulatory, laryngeal, and respiratory systems. This can primarily be attributed to difficulties in timing and coordination (McClean and Runyan, 2000; Kleinow et al., 2001; McClean et al., 2004; Max and Gracco, 2005; Loucks and De Nil, 2006; Loucks et al., 2007). Functional imaging studies have identified increased activity in the right pars opercularis, absence of bilateral auditory cortices, excessive cerebellar vermis activity, and increased dopaminergic midbrain activity in individuals who stutter (Sommer et al., 2002). There is also evidence of reversed activation of speech-related areas, with the motor cortex recruited early and the inferior frontal gyrus activated later during stuttering (Salmelin et al., 2000).

Of particular interest are potential neural abnormalities present during resting state, in the absence of overt stuttering behaviors. The resting state of the brain, also known as intrinsic or default mode, refers to the spontaneous activity that occurs when a person is not actively engaged in a task.

Spontaneous low-frequency oscillations (<0.1 Hz) of neural activity during rest reflect intrinsic functional connectivity networks in the brain and are thought to play a crucial role in various cognitive processes (Biswal et al., 1995). Aberrant resting-state functional connectivity has been reported in AWS compared to fluent controls, suggesting fundamental network coordination deficits (Chang and Zhu, 2013).

The frontal lobe of the brain contains an area called Broca’s area, which is important for expressive speech. It is linked to both language and nonlinguistic event sequencing in the left dorsal pars opercularis. Sequencing linguistic and nonlinguistic events is linked to the left dorsal pars opercularis, whereas sequencing articulatory events is linked to the ventral pars opercularis (Price, 2010). The SMA functions as an additional “neural marker” of developmental stuttering by integrating data from several brain circuits and managing information about motor programs, such as self-initiated motions and motor sequences (Busan, 2020).

Finally, investigating anomalies in functional and oscillatory dynamics during rest can elucidate the neurophysiological underpinnings of stuttering beyond overt speech behaviors. In addition, understanding the neural mechanisms underlying stuttering is crucial for developing effective therapeutic interventions and improving the overall quality of life for individuals who stutter.

The findings show that stutterers have higher activation in the right DLPFC, regardless of anticipation, and that anticipated words are linked to increased activation in the right DLPFC (Jackson et al., 2022).

In word retrieval, the left middle frontal cortex is implicated (Gabrieli et al., 1998). In the cortical speech and language network, an area for integrating information involving sensory modalities for semantic processing, the angular gyrus (AG) is essential (Daroff and Aminoff, 2014). It is connected to language, recall of memories, and focus. It is important for conveying visual information to Wernicke’s domain so that words perceived visually can have meaning (Seghier, 2013). Visual perception, multimodal sensory integration, and linguistic and semantic memory processing are all supported by the inferior temporal gyrus (ITG) sub region (Onitsuka et al., 2004).

Abnormalities in these regions could provide evidence for deficits in cognitive control, semantic processing, and speech perception pathways, respectively, underlying stuttering. This study aimed to examine both functional connectivity and spectral power differences during resting state between AWS and adults who do not stutter (AWNS) in the dorsolateral prefrontal cortex (DLPFC), AG, and ITG. In this study, we seek to find answers to two research questions: (1) how does the power of frequency spectra in the speech-related regions and associated areas of the brain at rest differ between AWS and AWNS? and (2) what are the differences in functional connectivity between speech-related regions and associated areas of the brain at rest between AWS and AWNS?

## Materials and methods

### Participants

The participants included 15 AWS with a mean age of 30.93 ± 8.45 years (3 females) and 15 AWNS with a mean age of 30.73 ± 8.30 years (3 females). The sample of AWS and AWNS were matched in terms of age and educational level. A *t*-test indicated that the groups did not significantly differ in age (*p* > 0.7). AWS recruited from various sources through convenience sampling, including private speech-language clinics, and support groups for individuals who stutter, all located in Tehran City, Iran. All AWS had received a diagnosis of stuttering from an expert speech and language clinician before their involvement in the current research. The inclusion criteria for AWS were as follows: (1) adults with history of persistent developmental stuttering, (2) aged between 18 and 45 years, (3) native speakers of Persian, (4) normal hearing and normal or near-normal eyesight, (5) no documented significant medical history, especially neurological conditions, (6) no other speech and language disorders and, (7) not taking any medication that affects brain functions, such as anti-anxiety or, anti-stress, anti-depressants.

Ten AWS were taken out of the study during project implementation. Four were on anti-anxiety medicine, and one had developed a stutter. Three subjects declined to take part in the study: one had psoriasis, one had migraines, and one had severe head motions and stuttering. Within the AWNS group, two participants stopped the study, while eight participants withdrew owing to psoriasis, panic disorder, migraine, and anorexia nervosa.

The exclusion criteria for both groups were if they were in a nervous condition during the study. Ethical approval for this study was obtained from the Tarbiat Modares University ethical council (IR.MODARES.REC.1401.099), and all participants provided informed consent by signing a consent form (Table 1).

**TABLE 1:** Stuttering severity and demographics in two groups (AWS and AWNS).

No	Age/AWNS	Gender/AWNS	Age/AWS	Gender/AWS	Stuttering Severity Instrument SSI-4	Stuttering severity
1	19	F	21	F	27.0	Moderate
2	40	M	41	M	30.5	Moderate
3	38	M	37	M	25.5	Moderate
4	30	M	31	M	35.5	Severe
5	25	F	25	F	29.5	Moderate
6	30	M	28	M	29.5	Moderate
7	38	F	39	F	24.5	Moderate
8	37	M	38	M	28.0	Moderate
9	39	M	40	M	31.0	Moderate
10	22	M	21	M	25.5	Moderate
11	27	M	27	M	26.0	Moderate
12	45	M	45	M	30.0	Moderate
13	29	M	28	M	28.0	Moderate
14	23	M	25	M	24.0	Moderate
15	19	M	18	M	29.0	Moderate
Mean	30.73		30.93		28.23	
SD	8.30		8.45		2.87	

### Procedure

The recent study was conducted in two sessions. In the first session, AWS was assessed to determine the severity of their stuttering (using the stuttering Severity Instrument–Fourth Edition: SSI-4). The procedure involved two speech tasks–a reading task and a spontaneous speech task. For the reading task, AWS read aloud a standardized 350-syllable Persian passage, allowing the researcher to evaluate their speech during a structured reading activity. The passage content focused on comparing behaviors in interpersonal interactions. Following this, participants engaged in a 3-min conversation with the examiner on a familiar topic of their choice. Open-ended prompts were utilized to encourage expressive responses, covering diverse topics. Examples of the prompts included: (1) share insights regarding your occupation (or studies), describe a typical day, (2) provide a detailed account of a recent vacation or trip, describe the destination and activities, (3) summarize the plot and characters of a favorite movie or book. What elements did you find enjoyable? (4) outline your hobbies and interests, describing your prefer free time activities, and (5) reflect on family or friends, sharing memorable experiences.

This spontaneous speech sample allowed researchers to assess the fluency of their speech during a more natural, unscripted interaction. The spontaneous speech samples were approximately 400 syllables in length. The Persian version of the SSI-4 was validated by Tahmasebi et al. (2018) and used reliably by trained speech-language pathologists (SLPs) for the current study. Additionally, an experienced SLP confirmed through interviews that the participants did not stutter. In the second session, electrical brain recordings were taken from all participants.

The data collected for this study is not currently available in a public or institutional repository but can be made available upon data analysis.

### EEG data collection and pre-processing

During the recording session, each participant was seated in a comfortable chair in a well-lit room. Resting-state EEG was recorded during eyes-opened rest (EOR), a state in which participants were instructed to keep their eyes open without engaging in any specific tasks. EEG was recorded for 5 min from each participant while they minimized head movement and blinking.

Electroencephalography data were recorded using a 64-channel brain system (G.tec Medical Engineering GmbH, Graz, Austria) at a sampling rate of 256 Hz and 16-bit resolution. Ag/Agcl electrodes were placed in 21 locations based on the conventional 10–20 International system (Fp1, Fp2, F1, F2, F3, F4, F5, F7, F8, AFZ, AF7, FC1, FC2, FC5, FC6, FT7, FT8, P3, P4, P9, and P10).

The ground and reference electrodes were attached to the forehead and right auricle, respectively.

To measure eye movement EOG channels were positioned open to the eyes as described by Boux et al. (2021). Electrodes for lateral eye movement recording were placed 1 cm above the left external canthus and 1 cm below the right external canthus and electrodes for vertical eye movement recording were placed under the supra and infraorbital crests. There were two sites for Electromyography (EMG): EMG artifact of the lips was recorded using bipolar connected electrodes on the left and lower and upper lips (oris and orbicularis) (Brooker and Donald, 1980). Impedance was maintained below 10K throughout the recording session.

Electroencephalography signals were band-pass filtered offline between 1 and 40 Hz. Stereotyped artifacts caused by muscle action were eliminated by discarding epochs. Muscle artifacts were removed by identifying spectral peaks that corresponded with muscle activation. Automated methods was performed based on independent component analysis, as implemented in the EEGLAB toolbox by Delorme and Makeig (2004).

### Analysis of neural oscillations

A total of 5 min of EEG signals were recorded for each participant. After preprocessing steps including noise cancellation, manual cleaning, and independent component Analysis (ICA) to reduce artifacts, the remaining 240 s of clean EEG data were retained for further analysis (Salimi et al., 2022). EEG data were preprocessed using the EEGLAB toolbox (Delorme and Makeig, 2004) in MATLAB v2021 (Math Works Inc., Natick, MA). Initially, visual inspection was conducted to identify and remove artifacts from the data. Subsequently, the independent component analysis (ICA) technique was applied using EEGLAB. ICA was employed in this study to identify components that were likely responsible for signal artifacts. By leveraging statistical properties and assuming underlying assumptions, ICA can effectively separate mixed oscillations and determine their origins (Hyvärinen, 2013). This analysis generated power spectra and topographic plots displaying the distribution of component values across the scalp. Components displaying artificial oscillations and mechanical noise were identified and removed based on recommended artifact rejection criteria.

The components display artificial oscillations and mechanical noise (Naik and Kumar, 2011). ICA has been widely utilized to eliminate EEG artifacts during baseline recordings, including artifacts related to the eye, blinks, eye movements, line noise, and heart activity (Tran et al., 2004). Each trial epoch was normalized, and the instantaneous power within five EEG frequency bands (delta: 1–4 Hz, theta: 4.5–8 Hz, alpha: 8.5–13 Hz, beta1: 13.5–30 Hz, gamma: 30.5–40 Hz) was extracted using a 4th-order Butterworth filter. Power analyses were conducted independently for specific brain regions including premotor and SMA (BA6), Broca’s region (BA44, BA45, and BA47), frontal area (BA8, BA9, and BA10), AG (BA39), and inferior temporal gyrus (BA20) within each frequency band. The frequency band powers of EEG signal were calculated from each region of interest using the band power function in MATLAB.

### Functional connectivity

Functional connectivity refers to the coherence and phase synchronization between different spatial locations in time series data. We used magnitude-squared coherence (MSC) function in MATLAB (mscohere) to estimate the coherence spectra between time series signals from each region of interest (Gholami-Mahtaj et al., 2022). The magnitude-squared coherence values range from 0 to 1 as a function of frequency, indicating the degree of correspondence between two time series x and y at each frequency. Higher values represent stronger functional connectivity. The magnitude-squared coherence (known as coherence) is a function of the power spectral densities, *P*_*xx*_(*f*) and *Pyy*(*f*), and the cross power spectral density, *P*_*xy*_(*f*) between x and y signal.


$$\begin{array}{c} C_{xy}\left(f\right)\;=\;\frac{|P_{xx}\left(f\right){|}^{2}}{P_{xx}\left(f\right)P_{yy}\left(f\right)}\\ \left(\text{Stoica and Moses, 2005; Malekpour et al., 2018}\right) \end{array}$$

### Statistical analysis

Statistical analysis was performed using Graph Pad Prism v8.0 software (Graph Pad Computer software, San Diego, CA). The Shapiro-Wilk test was used to assess the normality of the data. Because there was no normal distribution in the AWS, the non-parametric Mann-Whitney test was used to compare both groups. A *p*-value less than 0.05 was considered statistically significant. To account for multiple comparisons, the significance level (*p*-value) was corrected using a false discovery rate (FDR) procedure (Benjamini et al., 2001). We demonstrate the maximum effectiveness of the suggested hierarchical FDR method. This method revealed significant synchrony effects in the expected regions at an acceptable error rate of 5% (Hu et al., 2014).

## Results

### Frequency power analysis results

**FIGURE 1:**
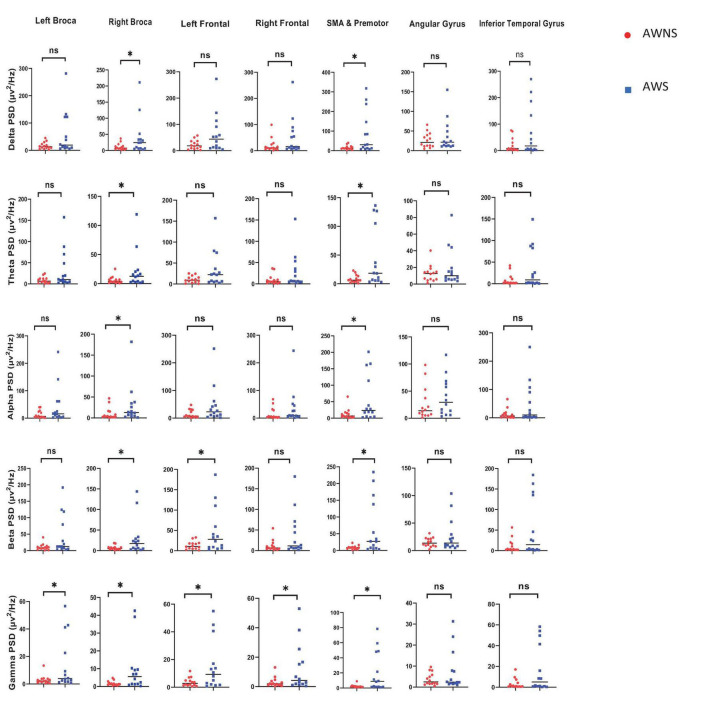
The present investigation aimed to explore the frequency power in various frequency bands, including alpha, beta, delta, theta, and gamma, within the central nervous system. The study compared two groups, namely adults who do not stutter (AWNS) and adults who stutter (AWS) in seven different speech regions. Statistical significance was set at *P* < 0.05 to determine the significance of the findings. The AWNS group was represented by red circles, while the patient group was represented by blue squares. ns, non-significant. *P < 0.05.

The results of frequency power analysis showed a significant increase in the gamma and beta band powers in the motor speech and frontal areas. Additionally, there was an increase in alpha, beta, delta, theta, and gamma band powers in the premotor and SMA regions, as well as in the alpha, delta and theta band powers in the right motor speech region in AWS compared to AWNS. In other words, AWS exhibit elevated activity across multiple frequency bands within the SMA, including increased power in the alpha, beta, delta, theta, and gamma bands. Furthermore, in the right motor speech area, AWS showed increased power spectral density specifically for the alpha, delta, and theta bands compared to controls. Moreover, both the beta and gamma bands in frontal and motor speech regions were higher in AWS compared to controls (*P* < 0.05), underscoring the substantial influence of beta and gamma band activity in AWS. Our findings regarding higher frequency band powers are consistent with results from FDR analysis, which also showed significantly increased power across frequency bands in AWS (*P*_*FDR*_ < 0.05) (Figure 1).

### Functional connectivity results

**FIGURE 2:**
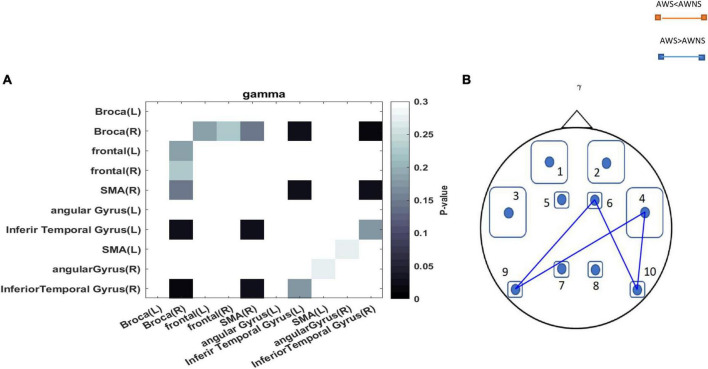
**(A)** The *p*-values obtained from comparing each pair of regions between the AWS and AWNS groups were represented in each cell. Darker colors in the plots indicated lower *p*-values. *p*-values less than 0.05 are considered statistically significant and were depicted in black. **(B)** A comparison between adults who stutter and adults who do not stutter regarding the coherence of the pairwise regions (*p* < 0.05). A blue line represented the coherency of the mentioned regions in individuals who stutter, while a red line represented the coherency in individuals who do not stutter. The abbreviations used to denote the different regions are as follows: 1 = Frontal-Left; 2 = Frontal-Right; 3 = Broca-Left; 4 = Broca-Right; 5 = SMA-Left; 6 = SMA-Right; 7 = Angular Gyrus-Left; 8 = Angular Gyrus-Right; 9 = Inferior Temporal Gyrus-Left; 10 = Inferior Temporal Gyrus-Right.

The functional connectivity analysis between AWS and AWNS revealed differences in all frequency bands of neural activity. When investigating the neural phase coherence patterns in speech regions at rest, it was found that AWS exhibited altered patterns of coherence between the motor speech, premotor supplementary motor area SMA, AG, and ITG areas compared to AWNS. Specifically, it was observed that the coherence in the power of the gamma spectrum was higher in the AWS group. These findings indicate increased functional connectivity in the gamma band between the right SMA region and the left ITG and right SMA and right ITG areas in AWS. Additionally, a significant difference was observed in the mentioned band between the Broca’s area and left ITG and right ITG and Broca’s area. In this study, we performed an unpaired *t*-test to compare functional connectivity between different brain regions in two groups of individuals with AWS and AWNS. The statistical significance of the *p*-values obtained from the connectivity analyses (*P* < 0.05) was compared to the outcomes after applying false discovery rate (FDR) correction. Our findings show that the significant *p*-values from the connectivity analyses aligned with the results after FDR correction (*P*_*FDR*_ < 0.05) (Figure 2).

## Discussion

The current study aimed to examine functional connectivity and spectral powers during rest periods between AWS and AWNS.

### Frequency power analysis

Frequency power analysis revealed several key differences between AWS and AWNS. First the frequency power analysis showed significant increases in gamma and beta band powers in motor speech and frontal areas in AWS compared to AWNS. The motor speech areas implicated include cortical regions involved in speech planning and production, while the frontal areas are associated with cognitive control and inhibition. These high-frequency bands are associated with active cognitive processing and task-related activation (Kaiser and Lutzenberger, 2005). The increased gamma and beta powers in motor speech regions therefore suggests that AWS may have elevated baseline activation in these areas, even at rest.

Second, AWS showed elevated power across in all bandwidths in premotor and SMA, especially at Beta and gamma bands. This highlights the particular role of fast rhythms involved in top-down cognitive control which may be disrupted in AWS. The premotor cortex works closely with the motor cortex to plan and execute movements, while the SMA is involved in motor sequence control (Yip and Lui, 2019). The broadband spectral power enhancement again indicates heightened baseline neural activity in speech motor planning regions in AWS.

Previous research has identified unusual regional brain asymmetries in AWS as an important factor in stuttering (Wells and Moore, 1990). Specifically, AWS showed higher alpha power in the right anterior region and greater power in the left posterior region compared to fluent speakers (Wells and Moore, 1990). Our findings align with these results, demonstrating significant alpha power in the right premotor and supplementary motor regions in AWS. Overall, atypical lateralization of neural activity emerges as a key characteristic differentiating AWS from fluent speakers.

Third, AWS exhibited greater delta and theta power in the right motor speech area. Slow wave delta and theta bands reflect decreased cortical activation (Gianotti et al., 2018). The laterality of this finding in the right motor cortex is notable, as left hemisphere frontal and motor areas typically dominate speech production (Price, 2010).

A positron emission tomography (PET) study conducted by Fox et al. (1996) supported the long-standing hypothesis that abnormal hemispheric lateralization is an fundamental cause of developmental stuttering. The study found increased activation in the right hemisphere of AWS. In addition, AWS exhibited heightened white matter volume (WMV) in the right hemisphere within key regions associated with speech production and language processing, including the superior temporal gyrus, precentral gyrus, and anterior middle frontal gyrus. In contrast, Penhune et al. (1996) observed leftward asymmetry of auditory cortex white matter in fluent controls. The increased right hemisphere WMV in stutterers may be attributed to atypical interhemispheric communication strategies (Craig-McQuaide et al., 2014). Overall, these findings support the notion that differences in right hemisphere speech and language processing play a role in developmental stuttering.

These findings align with previous research that has also identified abnormalities in resting-state brain activity in individuals who stutter.

It is important to note that impaired sensorimotor processing is considered the primary cause of stuttering. The contribution of the Theta group supports the idea that timing issues play a role in stuttering (Giraud and Poeppel, 2012). Delta waves play a role in modulating reaction times and are associated with anticipation and predictive coding. Speech entrained delta waves interact with distant brain networks that support lexical and semantic functions (Eggermont, 2021).

The changes observed in beta band power also suggest cognitive processes like attention are involved in stuttering, with differences in beta activity during rest indicating potential cognitive processing differences (Ofoe et al., 2018). However, maintaining fluent speech likely involves a complex interplay between sensorimotor and higher-order cognitive abilities (Jackson et al., 2015; Bowers et al., 2018).

Together, these results paint an overall picture of altered neural activity in key speech regions in AWS at rest. The combination of elevated fast-wave power suggesting hyper-excitability, along with slow-wave power indicating hypo activity, implies complex spectral disruption across the speech network in AWS.

### Functional connectivity

In addition to differences in frequency power, our analysis of functional connectivity also revealed aberrant neural dynamics in AWS. Specifically, we investigate phase coherence and connectivity patterns in the delta, theta, alpha, beta and gamma frequency bands between speech regions at rest in AWS compared to AWNS. Specifically, AWS showed an increase in coherence of the gamma band power between motor speech, SMA, and ITG areas. These findings confirm past work showing AWS exhibit greater functional connectivity compared to AWNS in resting-state brain activity (Jackson et al., 2015). Altered blood flow and functional connectivity between motor, language, auditory, and cognitive regions have also been demonstrated in AWS during rest (Jackson et al., 2019). Variations in speech-motor planning in stuttering further support connectivity abnormalities (Sengupta et al., 2017).

In particular, AWS exhibited increased functional connectivity in the alpha, delta and theta bands between right and left ITG regions and right Broca’s area. Increased delta connectivity was also seen between the ITG and Broca’s regions, as well as between the SMA and left and right ITG areas. This aligns with the view that abnormalities in phase coherence between frequency bands reflect miscommunication in the speech-motor network. The differences connectivity selectively in the delta, and anomalies in phase coherence pattern in the gamma bands during rest aligns with prior work and suggests dysfunctional integration of slow cortical rhythms in AWS. Our results are consistent with these previous studies of aberrant resting brain function in AWS (Sengupta et al., 2017). One possibility is that compensation by increased connections among regions is not necessary during rest (Lu et al., 2012).

Xuan’s study further demonstrated a reduced resting-state functional connectivity between the left IFG and the right IPL, along with increased functional connectivity in the sensorimotor network, among AWS (Xuan et al., 2012).

In contrast, significant between-group differences in functional connectivity were found for other frequency bands, including alpha, delta and theta oscillations (Joos et al., 2014). Delta waves are associated with cognitive and motor control, and greater coherence facilitates communication across brain networks (Başar et al., 2001; Harmony, 2013). Our findings indicate AWNS have more coordinated low-frequency and high-frequency oscillations interactions between key speech regions including the motor cortex, AG, and ITG areas. In contrast, AWS shows a decoupling of these regions, reflecting reduced synchrony during rest.

Taken together, the extensive reductions in both spectral power and inter-regional functional connectivity across the delta, theta, and faster rhythms provide converging electrophysiological evidence for aberrant neural dynamics in core speech regions in AWS, even without overt speech demands.

The dysfunctional cortical oscillations likely contribute to impaired sensorimotor processing and cognitive-linguistic deficits. These fundamental neurophysiological differences may form the core deficit in stuttering. Further research can elucidate the relationship between resting state connectivity patterns and spectral anomalies during active speech and language tasks. This may lead to neurophysiological markers that predict persistence and recovery in developmental stuttering.

## Conclusion

The broad electrophysiological differences during simple rest indicate that functional abnormalities in speech networks persist even in the absence of overt stuttering demands. This supports the view that stuttering stems from core neurophysiological deficiencies, rather than solely situation-specific anxiety or cognitions. Further research can build on these foundational spectral findings to elucidate neural oscillatory dynamics during speech versus rest and their relationship with stuttering severity.

### Limitations and future suggestions

This study provided valuable insights into the role of neural coherence in stuttering but had limitations that should be addressed in future research. The relatively small sample size, though statistically adequate, constrains generalizability. Future studies should include larger, more diverse samples, particularly varying in stuttering severity.

Our findings contribute physiological evidence that even at rest, AWS shows fundamental neural activation differences related to speech. Further research should explore how these intrinsic patterns interact with active speech to precipitate stuttering events, which may inform more targeted treatments. Additional work is needed to clarify the specific neural mechanisms underlying the identified connectivity differences and their implications for interventions. Larger, more diverse samples should be included. Critically, exploring relationships between resting state anomalies, frequency power differences, and stuttering during speech is key to explaining why AWS can speak fluently at times but stutter at others. Elucidating these network dynamics represents an important direction for stuttering research.

## Data availability statement

The original contributions presented in this study are included in the article/supplementary material, further inquiries can be directed to the corresponding authors.

## Ethics statement

The studies involving humans were approved by the Tarbiat Modares University Ethical Council (IR.MODARES.REC.1401.099). The studies were conducted in accordance with the local legislation and institutional requirements. Written informed consent for participation in this study was provided by the participants’ legal guardians/next of kin.

## Author contributions

AV: Formal analysis, Methodology, Writing—original draft. SB: Software, Writing—review and editing. AG: Supervision, Writing—review and editing. GG: Supervision, Writing—review and editing. HA: Writing—review and editing. MR: Conceptualization, Methodology, Project administration, Writing—review and editing.
